# Supplementary material for: Enhanced astrocyte responses are driven by a genetic risk allele associated with multiple sclerosis
Source: Nat Commun. 2018 Dec 17;9:5337. doi: 10.1038/s41467-018-07785-8 (PMC6297228; doi:10.1038/s41467-018-07785-8)
Supplement: Supplementary file 1 — Supplementary Information [file 41467_2018_7785_MOESM1_ESM.pdf]

Enhanced Astrocyte Responses are Driven by a Genetic Risk Allele Associated with Multiple Sclerosis

Supplementary Information

Ponath et al.

Supplementary Figure 1.

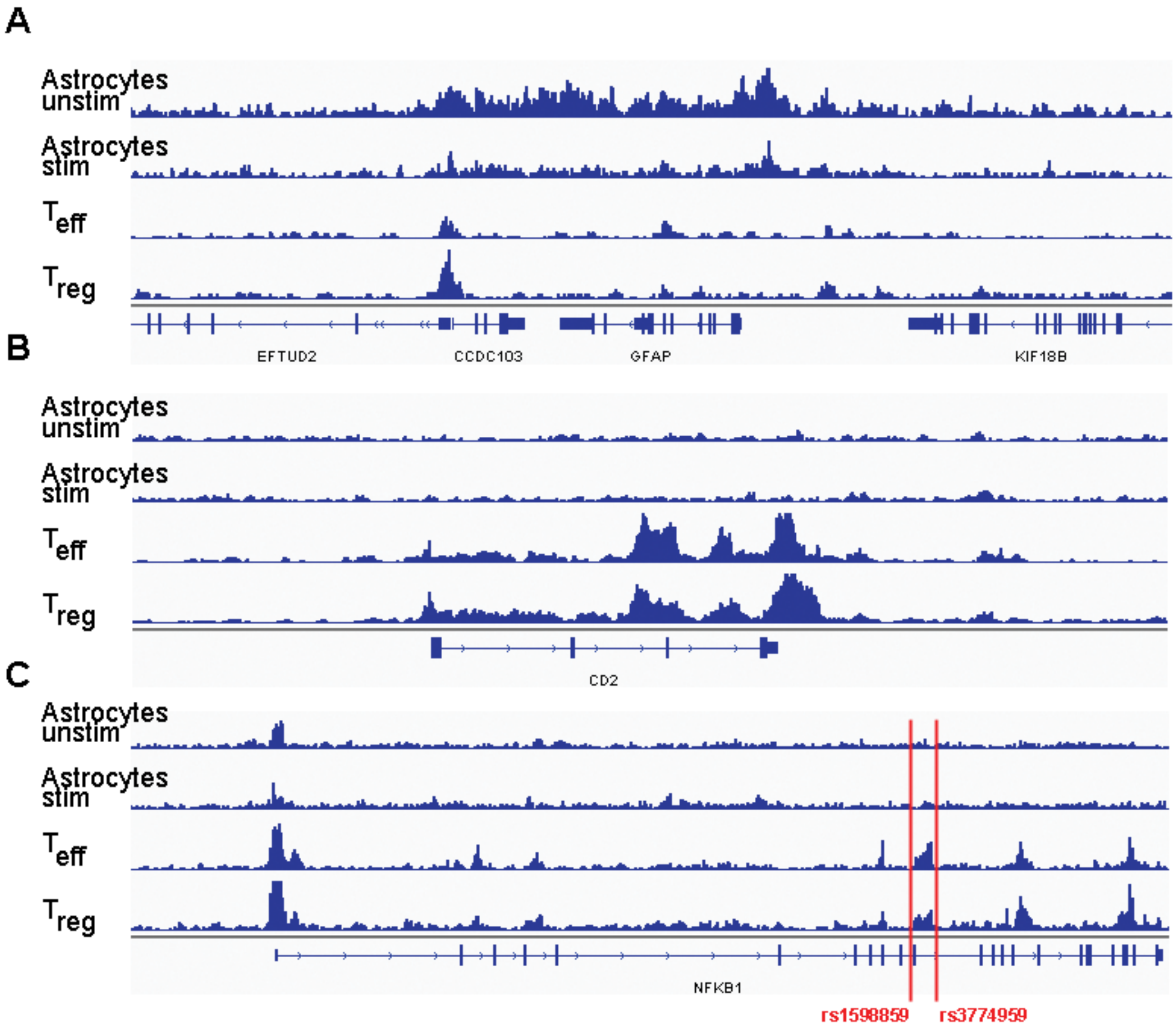

Supplementary Figure 1. Comparison of normalized ATAC-seq profiles between GLAST-purified unstimulated and stimulated human fetal astrocytes and *ex vivo* unstimulated T cells. ATAC-seq profiles near the (**A**) *GFAP*, (**B**) *CD2* and (**C**) *NFKB1* loci. (**A**) Note the increased chromatin accessibility in the vicinity of the astrocyte lineage-specific *GFAP* gene in astrocytes but not in T cells. (**B**) The chromatin region around the T cell specific gene *CD2* is accessible in T cells but not astrocytes. (**C**) Chromatin landscape of *NFKB1* with high DNA accessibility in a haplotype block containing the tagging SNPs rs3774959 and rs1598859 in T cells but not astrocytes. The variants confer risk for inflammatory bowel disease and systemic sclerosis, respectively, but not for MS.

Supplementary Figure 2.

MS Patient	Variant	Age	Gender	Disease onset (age)	Disease course	Treatment at time of biopsy	Healthy Donor	Variant	Age	Gender
1	risk	54	Female	29	SPMS	Rituximab	1	risk	65	Female
2	risk	45	Female	35	RRMS	Natalizumab	2	risk	28	Female
3	risk	42	Female	23	RRMS	Natalizumab	3	risk	71	Male
4	protective	56	Female	33	SPMS	Natalizumab	4	protective	37	Female
5	protective	47	Female	32	RRMS	Rituximab	5	protective	35	Female
6	protective	26	Female	22	RRMS	Natalizumab	6	protective	32	Female

Supplementary Figure 2. Clinical data and demographics of MS patients and healthy donors without neurological disease from whom skin biopsies were obtained for generation of iPSC lines.

Supplementary Figure 3.

		iPSC lines						MS Cases					iPSC lines						MS Cases				
**gene**	**SNP**	1	2	3	4	5	6	1	2	3	4	5	7	8	9	10	11	12	6	7	8	9	10
NFKB1	rs7665090	risk	risk	risk	risk	risk	risk	risk	risk	risk	risk	risk	prot	prot	prot	prot	prot	prot	prot	prot	prot	prot	prot
TNFRSF1A	rs1800693	risk	het	het	het	het	het	prot	het	het	het	het	het	het	risk	het	het	het	het	het	het	het	risk
LTBR	rs12296430	het	het	het	het	het	het	het	het	het	het	het	het	het	risk	het	het	het	het	het	het	het	het
TNFSF14A	rs1077667	het	risk	het	het	het	het	het	prot	het	het	het	prot	het	het	het	het	het	het	het	het	het	het
TRAF3	rs12148050	het	het	het	het	prot	het	het	het	het	risk	het	het	het	het	prot	het	het	het	het	risk	het	het
CD40	rs4810485	het	het	het	risk	het	het	het	het	het	prot	het	risk	het	het	het	het	het	het	het	het	prot	het

Supplementary Figure 3. Genotypic profiles of iPSC lines and MS tissue for NF-κB relevant MS risk variants. For clarity, genotypes are reported as risk (red), heterozygous (het, yellow), and protective (prot, green), regarding their association with MS susceptibility. iPSC lines and MS cases of both groups (rs7665090^{GG} and rs7665090^{AA}) show random distribution of potentially confounding genetic variants.

Supplementary Figure 4.

A

rs7665090	protective	risk
Gene	Fold Change	Fold Change
BIRC3	7.12	3.16
C3	unchanged	2.02
CCL2	4.61	10.38
CCL5	5.63	8.02
CD40	2.82	unchanged
CD69	3.73	16.96
CXCL1	11.14	7.98
CXCL10	226.79	201.61
CXCL2	11.2	8
CXCL9	2.83	4.01
GADD45B	unchanged	3.22
ICAM1	2.81	6.33
IL15	4.48	12.86
CXCL8	14.12	6.37
IRF1	8.9	16.13
LTA	unchanged	2.48
LTB	2.24	4.01
NFKB2	3.57	3.22
NFKBIA	4.46	4
NR4A2	2.78	unchanged
PLAU	4.42	3.19
RELB	11.25	7.95
SELE	5.72	5.11
STAT1	2.8	3.17
TNF	36.14	7.95
TNFRSF1B	unchanged	2.53
TNFSF10	unchanged	3.2
VCAM1	30.03	26.29
B2M	unchanged	2.54
RPLP0	unchanged	2.51

B

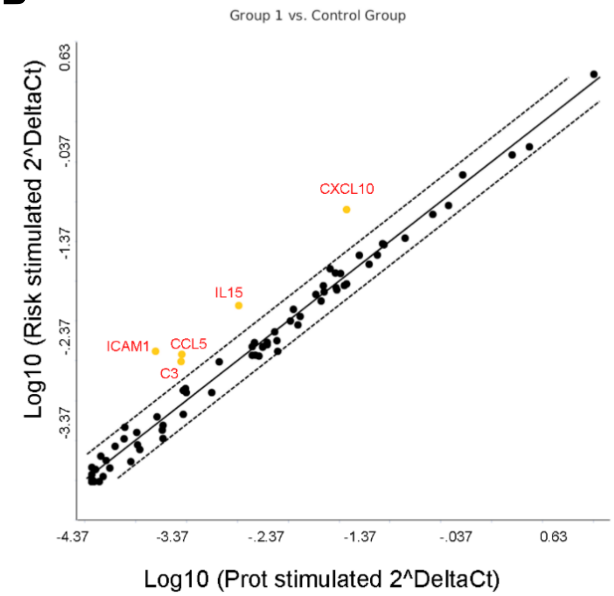

C

Gene Symbol	Fold Regulation	p-value	FDR p-value
C3	3.46	0.0004858	0.007047
CCL5	4.22	0.0002155	0.009396
CXCL10	6.66	0.0037238	0.014094
ICAM1	8.39	0.0011415	0.02481675
IL15	4.72	8.1E-05	0.0410118

D

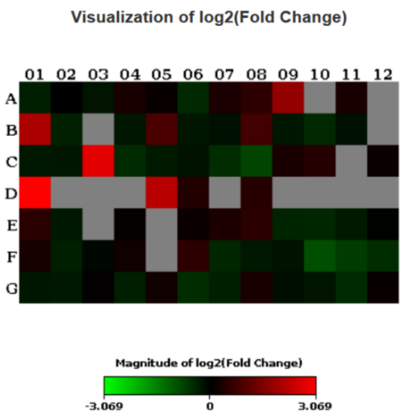

Layout	1	2	3	4	5	6	7	8	9	10	11	12
**A**	ADM<br>**-1.28**	AGT<br>**1**<br>B	AKT1<br>**-1.19**	ALDH3A2<br>**1.23**	BCL2A1<br>**1.07**<br>B	BCL2L1<br>**-1.39**<br>B	BIRC2<br>**1.24**	BIRC3<br>**1.45**<br>B	C3<br>**3.46**<br>A	CCL11<br>**-1.22**<br>C	CCL2<br>**1.23**<br>B	CCL22<br>**-1.22**<br>C
**B**	CCL5<br>**4.22**<br>A	CCND1<br>**-1.3**	CCR5<br>**-1.22**<br>C	CD40<br>**-1.21**	CD69<br>**1.87**<br>B	CD80<br>**-1.2**<br>B	CD83<br>**-1.15**	CDKN1A<br>**1.73**	CFB<br>**-1.2**<br>B	CSF1<br>**-1.39**	CSF2<br>**-1.14**<br>B	CSF2RB<br>**-1.22**<br>C
**C**	CSF3<br>**-1.22**<br>B	CXCL1<br>**-1.2**	CXCL10<br>**6.66**	CXCL2<br>**-1.42**	CXCL9<br>**-1.25**<br>B	EGFR<br>**-1.17**	EGR2<br>**-1.45**<br>B	F3<br>**-1.77**<br>B	F8<br>**1.22**<br>B	FAS<br>**1.36**	FASLG<br>**-1.22**<br>C	GADD45B<br>**1.09**
**D**	ICAM1<br>**8.39**<br>A	IFNB1<br>**-1.22**<br>C	IFNG<br>**-1.22**<br>C	IL12B<br>**-1.22**<br>C	IL15<br>**4.72**<br>A	IL1A<br>**1.34**<br>B	IL1B<br>**-1.22**<br>C	IL1R2<br>**1.36**<br>B	IL1RN<br>**-1.22**<br>C	IL2<br>**-1.22**<br>C	IL2RA<br>**-1.22**<br>C	IL4<br>**-1.22**<br>C
**E**	IL6<br>**1.39**<br>B	CXCL8<br>**-1.22**	INS<br>**-1.22**<br>C	IRF1<br>**1.03**	LTA<br>**-1.22**<br>C	LTB<br>**1.09**<br>B	MAP2K6<br>**1.27**<br>B	MMP9<br>**1.43**<br>B	MYC<br>**-1.35**	MYD88<br>**-1.36**<br>B	NCOA3<br>**-1.25**	NFKB1<br>**-1.02**
**F**	NFKB2<br>**1.2**	NFKBIA<br>**-1.29**	NQO1<br>**-1.06**	NR4A2<br>**1.15**	PDGFB<br>**-1.22**<br>C	PLAU<br>**1.45**	PTGS2<br>**-1.36**<br>B	REL<br>**-1.23**	RELA<br>**-1.16**	RELB<br>**-1.91**	SELE<br>**-1.65**	SELP<br>**-1.45**<br>B
**G**	SNAP25<br>**-1.19**<br>B	SOD2<br>**-1.22**	STAT1<br>**1.02**	STAT3<br>**-1.29**	STAT5B<br>**1.14**	TNF<br>**-1.44**	TNFRSF1<br>B<br>**-1.29**<br>B	TNFSF10<br>**1.23**<br>B	TP53<br>**-1.12**	TRAF2<br>**-1.18**	VCAM1<br>**-1.41**<br>B	XIAP<br>**1.06**

E

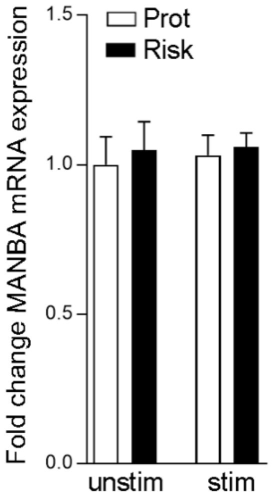

Supplementary Figure 4. Effect of the rs7665090 risk variant on human iPSC-derived astrocytes. (**A**) Upregulated NF-κB target genes from a 84 NF-κB target gene panel after stimulation with TNFα, IL-1β and IFNγ in astrocytes with the protective and risk variants compared to baseline (**B**, **C**) Scatter plot profiling astroglial expression of NF-κB target genes after stimulation with TNFα, IL-1β, and IFNγ. Red dots indicate genes with ≥2-fold expression. (**D**) Fold-change values greater than one indicate upregulation. A: This data means that the expression is relatively low in one sample and reasonably detected in the other sample, suggesting that the actual fold-change value is at least as large as the calculated. B: The relative expression level is low in both control and test samples, and the p-value for the fold-change is either unavailable or relatively high ($p > 0.05$). C: This generated average threshold cycle is either not determined or greater than the defined cut-off value (default 35) in both samples, meaning that its expression was undetected, making this fold-change result erroneous and un-interpretable. Data is visualized in a heat map for graphical representation of the PCR array results. (**E**) *MANBA* mRNA expression remains unchanged in iPSC-derived astrocytes after stimulation with TNFα, IL-1β, and IFNγ, and shows no differential gene expression according to rs7665090^{G} genotype (6 lines per group) revealed by quantitative real-time PCR.

Supplementary Figure 5. Clinical data of autopsied MS cases§

Patient	Variant	Age	Gender	Disease onset (age)	Disease course	PMI (hrs)	All lesions	Chronic active lesions (analysis of astrocyte phenotype)
1*	risk	32	female	26	RRMS	3.5	2	2
2*	risk	42	male	34	RRMS	8	1	1
3*	risk	38	male	26	RRMS	23	6	1
4*	risk	61	female	unknown	SPMS	11	3	1
5*	risk	50	female	34	SPMS	5	2	1
6#	risk	45	female	24	RRMS	4	3	0
7#	risk	60	female	42	PPMS	24	2	0
8#	risk	67	female	unknown	SPMS	9	2	0
9*	protective	72	female	45	SPMS	22	3	2
10*	protective	30	Male	25	RRMS	12	2	1
11*	protective	53	Male	unknown	SPMS	11	7	2
12*	protective	38	Male	32	RRMS	9	3	2
13*	protective	76	female	unknown	SPMS	3	1	1
14#	protective	71	Male	unknown	SPMS	13	2	0

* used for analysis of chemokines, infiltration with $CD3^+$ cells and lesion size (chronic active lesions)

# used for analysis of lesion size only (chronic silent lesions)

§ none of the MS patients from whom autopsy material was obtained were treated with highly-effective treatments such as natalizumab, alemtuzumab, ocrelizumab, or rituximab

Supplementary Figure 6.

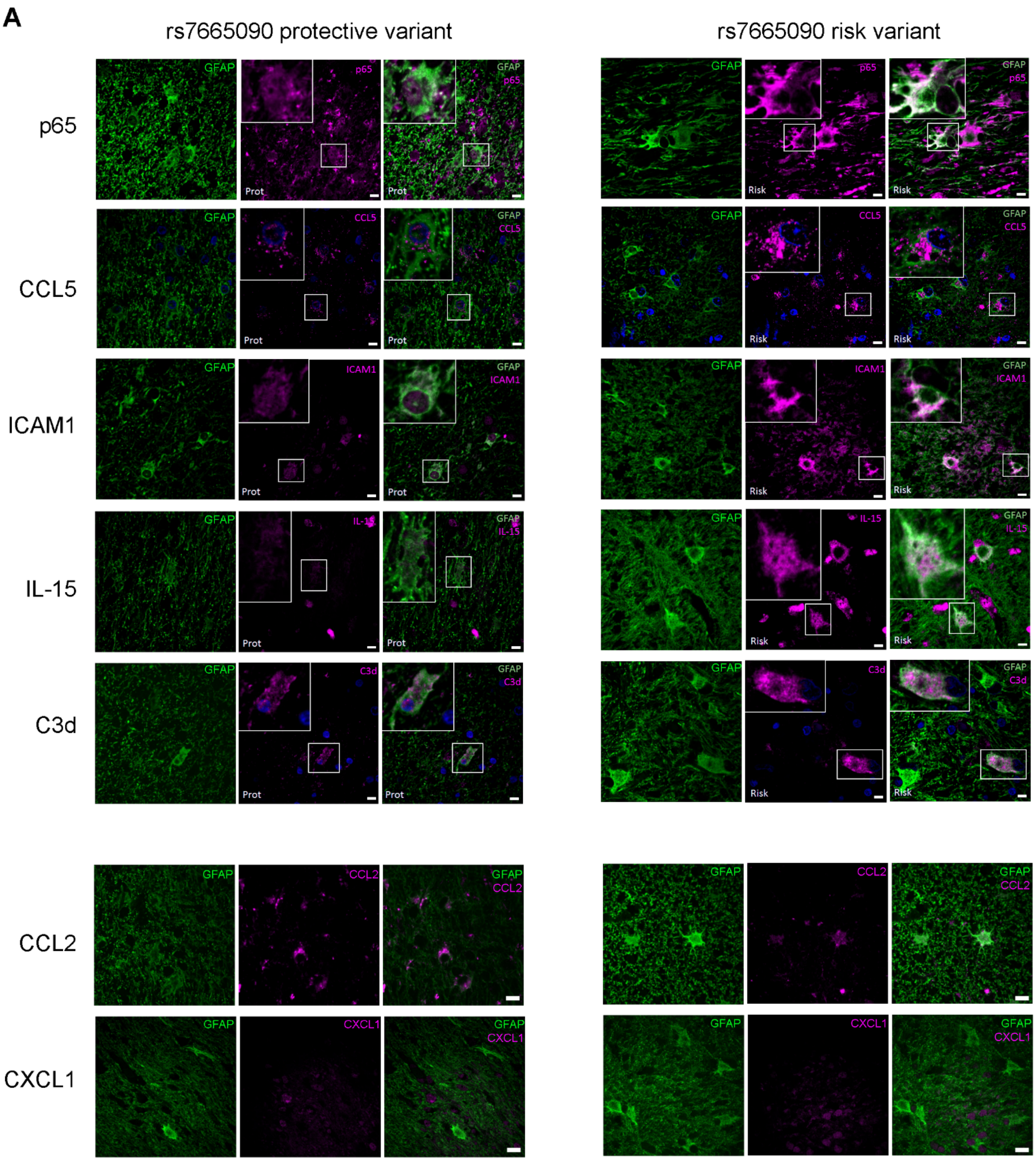

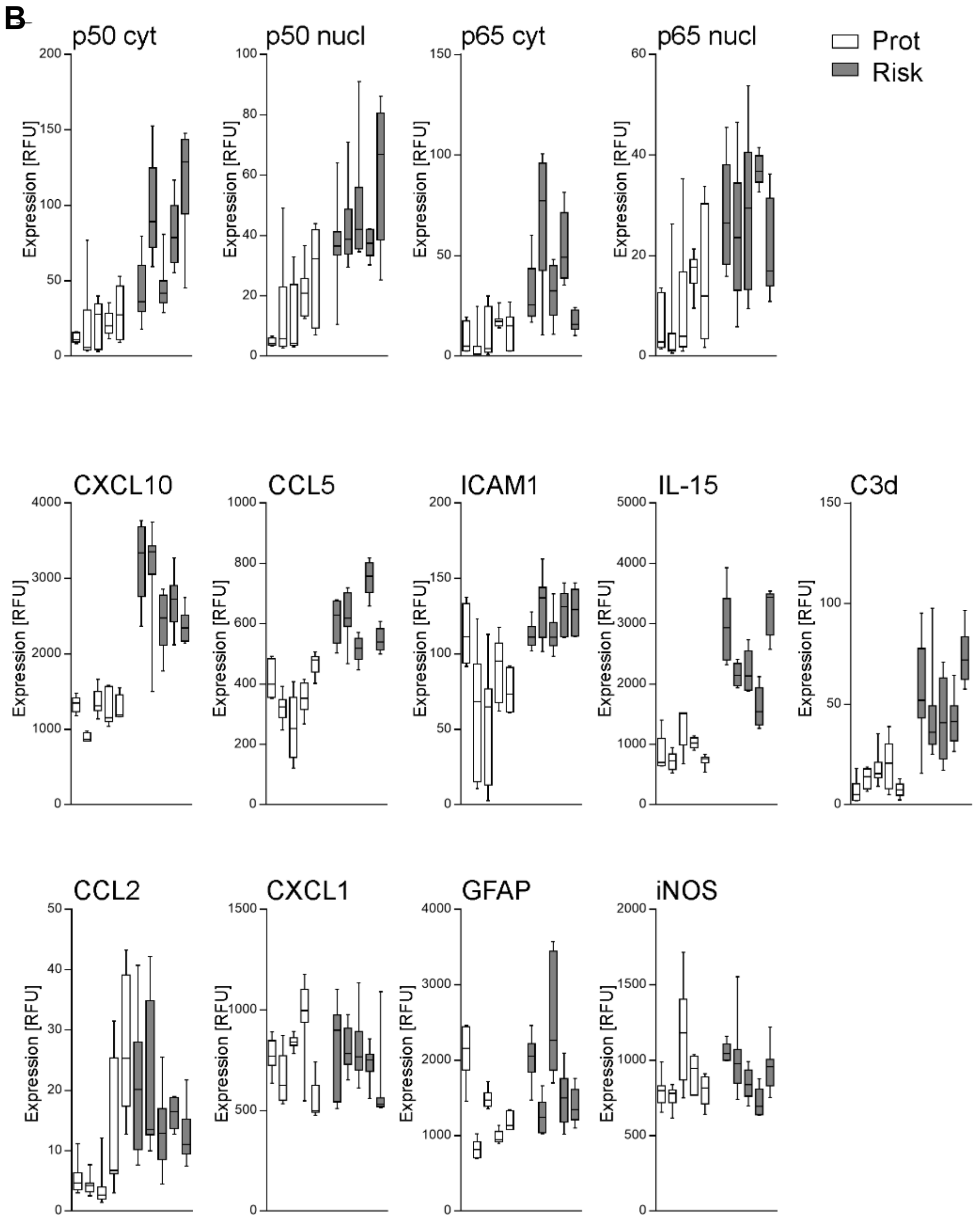

B
p50 cyt
p50 nucl
p65 cyt
p65 nucl
Prot
Risk
CXCL10
CCL5
ICAM1
IL-15
C3d
CCL2
CXCL1
GFAP
iNOS
Expression [RFU]

C

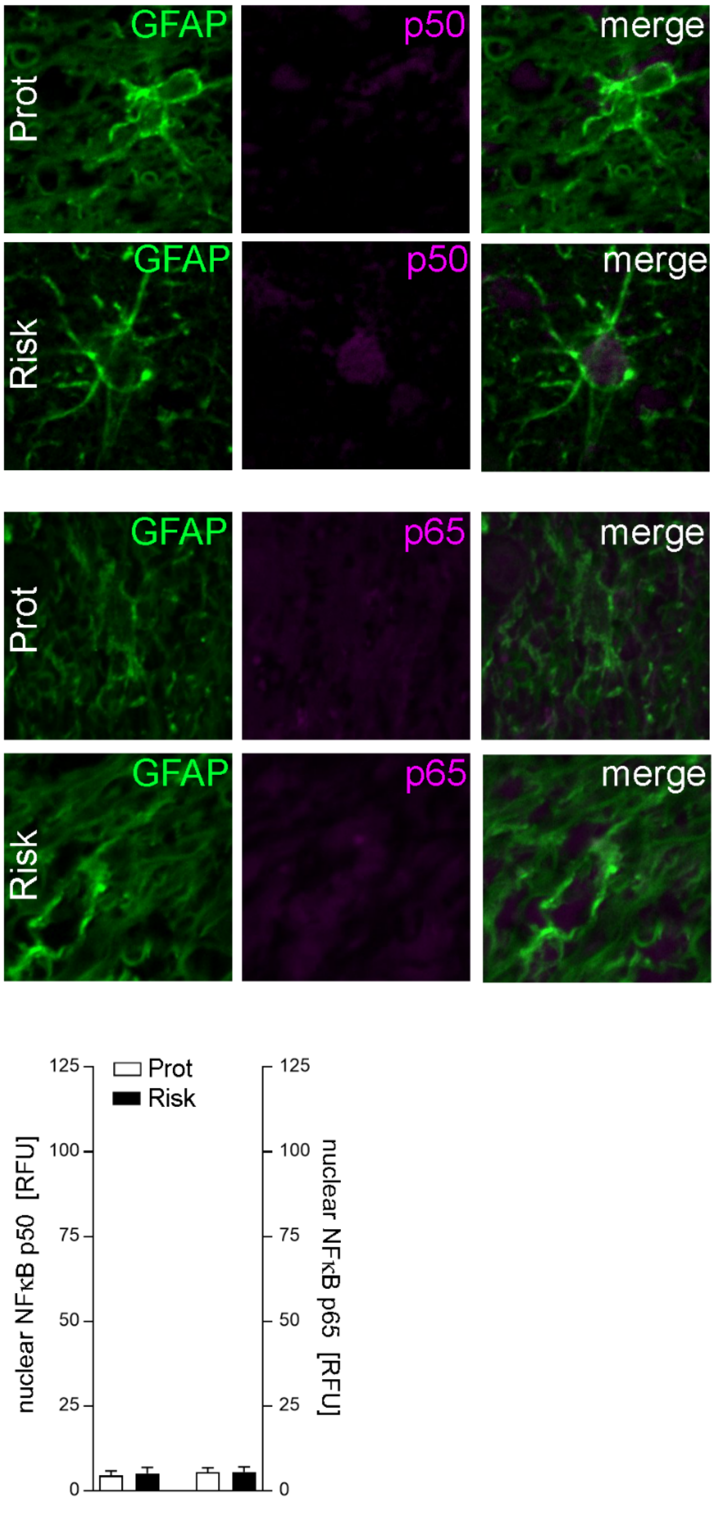
GFAP
p50
merge
Prot
Risk
p65
125
100
75
50
25
0
Prot
Risk
nuclear NFκB p50 [RFU]
nuclear NFκB p65 [RFU]

D

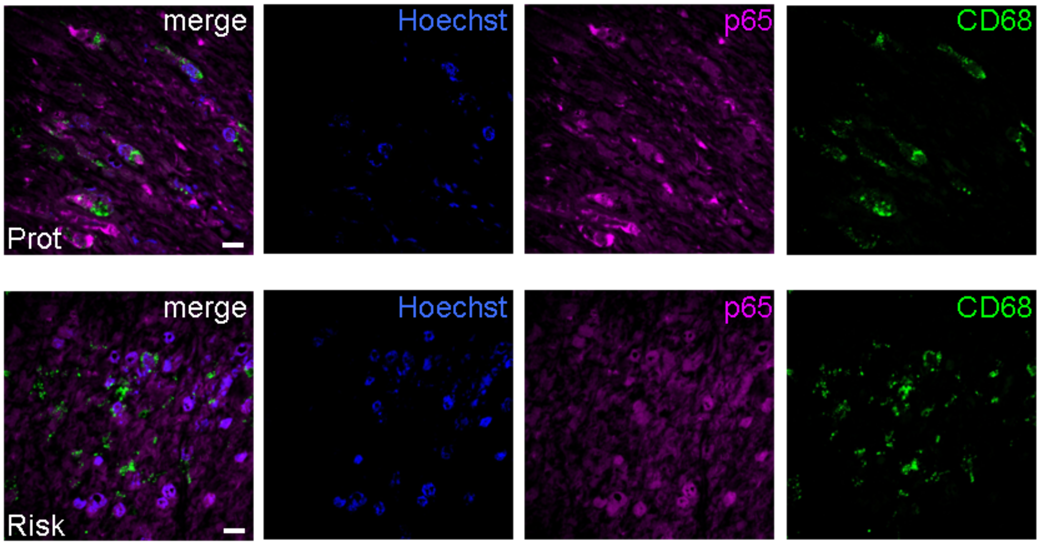
merge
Hoechst
p65
CD68
Prot
merge
Hoechst
p65
CD68
Risk

E

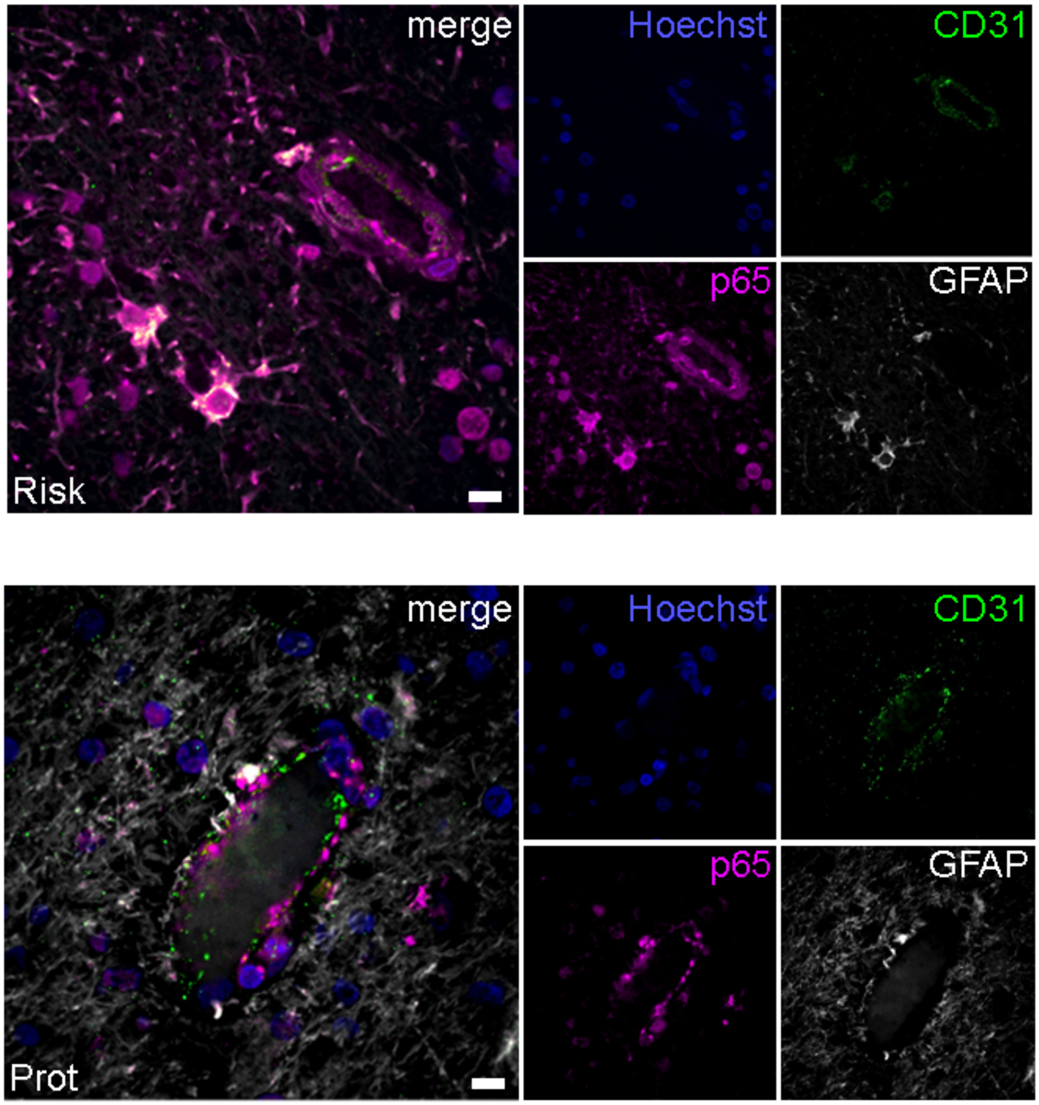
merge
Hoechst
CD31
p65
GFAP
Risk
merge
Hoechst
CD31
p65
GFAP
Prot

Supplementary Figure 6. Densitometric analysis. (**A**) Representative confocal microscopy images of hypertrophic astrocytes homozygous for the risk or protective variants located at the lesion edge, stained with fluorescent antibodies against GFAP (green) p65, CCL5, ICAM1, IL-15, C3d, CCL2, and CXCL1 (magenta), respectively. (**B**) Densitometric quantification of protein expression in the cytosol/nucleus of hypertrophic lesional astrocytes. (**C**) Representative confocal microscopy images of non-reactive astrocytes with the risk or protective variants, located in the normal appearing white matter (NAWM), stained with fluorescent antibodies against GFAP (green), p50 and p65 (both magenta). Densitometric quantification of p50 and p65 protein expression in the nucleus of astrocytes in NAWM. Each bar represents the average fluorescence from 10-20 astrocytes per case, with five cases per group (white = prot; gray = risk). (**D**) Representative confocal microscopy images of microglia cells carrying the risk or protective variants, located in the NAWM, stained with fluorescent antibodies against CD68 (green) and p65 (magenta). (**E**) Representative confocal microscopy images of endothelial cells carrying the risk or protective variants, located in the NAWM, stained with fluorescent antibodies against CD31 (green), p65 (magenta), and GFAP (gray). Scale bars = 15 µm.

Supplementary Figure 7.

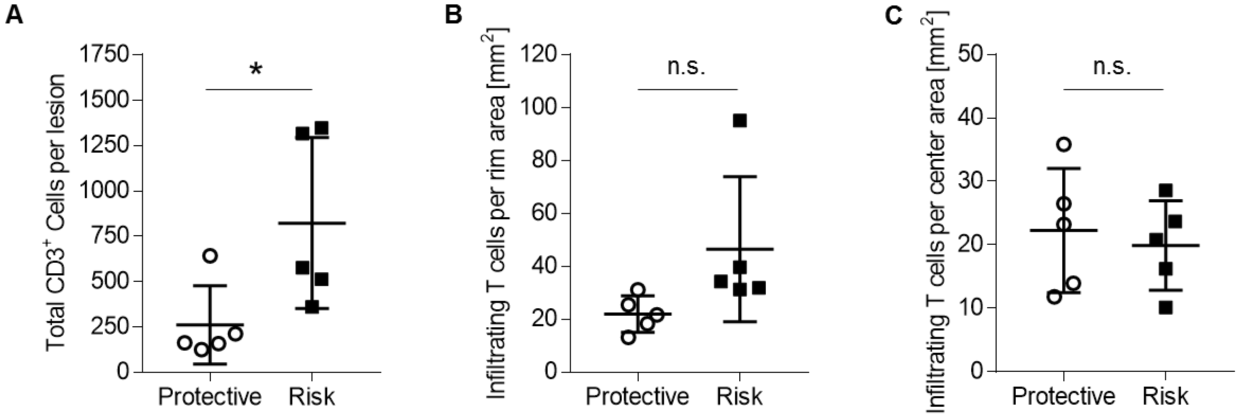

Supplementary Figure 7. Quantification of perivascular $CD3^+$ T cells in chronic active lesions with the risk or protective genotype. $CD3^+$ cells were counted in 29 lesions (15 risk group, 14 protective group) from 10 MS cases (5 cases per group), with 1 to 5 lesions per case. Each dot represents an average $CD3^+$ cell count from all lesions of a single case. **(A)** Total number of infiltrating $CD3^+$ cells in lesions with the risk or protective variants (p=0.0415). **(B)** Number of infiltrating $CD3^+$ cells in the lesion rim, normalized to rim area (p=0.0886). **(C)** Number of infiltrating $CD3^+$ cells in the lesion center, normalized to center area (p=0.6720). Data represents means ± s.d. p-values shown for unpaired student's t-test. *p<0.05.

Supplementary Figure 8.

A

P values	p50 Cytosol	p50 Nucleus	p65 Cytosol	p65 Nucleus	CXCL10	CCL5	C3d	ICAM1	IL-15	Lesion Area	CD3+ cells	CD68+ cells	CCL2	CXCL1	GFAP	iNOS
p50 Cytosol		0.0016	0.0443	0.0603	0.0225	0.0214	0.0044	0.0194	0.0159	0.0829	0.0054	0.1206	0.3436	0.4031	0.9421	0.8385
p50 Nucleus	0.0016		0.1238	0.0162	0.0156	0.0301	0.0008	0.0357	0.0025	0.0262	0.0305	0.0606	0.1525	0.4378	0.5526	0.8730
p65 Cytosol	0.0443	0.1238		0.0093	0.0045	0.0061	0.1645	0.0283	0.2322	0.7752	0.0177	0.4913	0.1542	0.5593	0.7143	0.9075
p65 Nucleus	0.0603	0.0162	0.0093		0.0024	0.0009	0.0282	0.0243	0.0619	0.3425	0.0598	0.3707	0.0891	0.7889	0.3216	0.8764
CXCL10	0.0225	0.0156	0.0045	0.0024		0.0017	0.0033	0.0072	0.0033	0.1104	0.0356	0.6086	0.1313	0.7565	0.2149	0.5052
CCL5	0.0214	0.0301	0.0061	0.0009	0.0017		0.0400	0.0024	0.0838	0.5666	0.1248	0.3730	0.0564	0.6526	0.4376	0.5159
C3d	0.0044	0.0008	0.1645	0.0282	0.0033	0.0400		0.0306	0.0000	0.0048	0.0533	0.3381	0.4292	0.8476	0.4585	0.4411
ICAM1	0.0194	0.0357	0.0283	0.0243	0.0072	0.0024	0.0306		0.0459	0.4267	0.1385	0.8686	0.2961	0.9403	0.2190	0.6464
IL-15	0.0159	0.0025	0.2322	0.0619	0.0033	0.0838	0.0000	0.0459		0.0009	0.0692	0.5167	0.4430	0.9321	0.2656	0.2456
Lesion Size	0.0829	0.0262	0.7752	0.3425	0.1104	0.5666	0.0048	0.4267	0.0009		0.0991	0.6340	0.7314	0.8413	0.4467	0.0411
CD3+ cells	0.0054	0.0305	0.0177	0.0598	0.0356	0.1248	0.0533	0.1385	0.0692	0.0991		0.2673	0.6916	0.9464	0.6643	0.3483
CD68+ cells	0.1206	0.0606	0.4913	0.3707	0.6086	0.3730	0.3381	0.8686	0.5167	0.6340	0.2673		0.4041	0.0541	0.4182	0.3942
CCL2	0.3436	0.1525	0.1542	0.0891	0.1313	0.0564	0.4292	0.2961	0.4430	0.7314	0.6916	0.4041		0.5569	0.9963	0.8998
CXCL1	0.4031	0.4378	0.5593	0.7889	0.7565	0.6526	0.8476	0.9403	0.9321	0.8413	0.9464	0.0541	0.5569		0.6126	0.2287
GFAP	0.9421	0.5526	0.7143	0.3216	0.2149	0.4376	0.4585	0.2190	0.2656	0.4467	0.6643	0.4182	0.9963	0.6126		0.8792
iNOS	0.8385	0.8730	0.9075	0.8764	0.5052	0.5159	0.4411	0.6464	0.2456	0.0411	0.3483	0.3942	0.8998	0.2287	0.8792	

Pearson r	p50 Cytosol	p50 Nucleus	p65 Cytosol	p65 Nucleus	CXCL10	CCL5	C3d	ICAM1	IL-15	Lesion Size	CD3+ cells	CD68+ cells	CCL2	CXCL1	GFAP	iNOS
p50 Cytosol		0.86	0.64	0.61	0.71	0.71	0.81	0.72	0.73	0.57	0.80	-0.52	0.34	-0.30	-0.03	0.07
p50 Nucleus	0.86		0.52	0.73	0.73	0.68	0.88	0.67	0.84	0.69	0.68	-0.61	0.49	-0.28	0.21	0.06
p65 Cytosol	0.64	0.52		0.77	0.81	0.79	0.48	0.69	0.42	0.10	0.72	-0.25	0.49	0.21	0.13	0.04
p65 Nucleus	0.61	0.73	0.77		0.84	0.88	0.69	0.70	0.61	0.34	0.61	-0.32	0.56	0.10	0.35	-0.06
CXCL10	0.71	0.73	0.81	0.84		0.85	0.83	0.78	0.82	0.54	0.67	-0.19	0.51	0.11	0.43	0.24
CCL5	0.71	0.68	0.79	0.88	0.85		0.65	0.84	0.57	0.21	0.52	-0.32	0.62	-0.16	0.28	-0.23
C3d	0.81	0.88	0.48	0.69	0.83	0.65		0.68	0.97	0.81	0.63	-0.34	0.28	-0.07	0.27	0.28
ICAM1	0.72	0.67	0.69	0.70	0.78	0.84	0.68		0.64	0.28	0.50	-0.06	0.37	0.03	0.43	-0.17
IL-15	0.73	0.84	0.42	0.61	0.82	0.57	0.97	0.64		0.88	0.60	-0.23	0.27	-0.03	0.39	0.40
Lesion Size	0.57	0.69	0.10	0.34	0.54	0.21	0.81	0.28	0.88		0.55	-0.17	0.12	-0.07	0.27	0.65
CD3+ cells	0.80	0.68	0.72	0.61	0.67	0.52	0.63	0.50	0.60	0.55		-0.39	0.14	0.02	0.16	0.33
CD68+ cells	-0.52	-0.61	-0.25	-0.32	-0.19	-0.32	-0.34	-0.06	-0.23	-0.17	-0.39		-0.30	0.62	0.29	0.30
CCL2	0.34	0.49	0.49	0.56	0.51	0.62	0.28	0.37	0.27	0.12	0.14	-0.30		-0.21	0.00	-0.05
CXCL1	-0.30	-0.28	0.21	0.10	0.11	-0.16	-0.07	0.03	-0.03	-0.07	0.02	0.62	-0.21		0.18	0.42
GFAP	-0.03	0.21	0.13	0.35	0.43	0.28	0.27	0.43	0.39	0.27	0.16	0.29	0.00	0.18		0.06
iNOS	0.07	0.06	0.04	-0.06	0.24	-0.23	0.28	-0.17	0.40	0.65	0.33	0.30	-0.05	0.42	0.06	

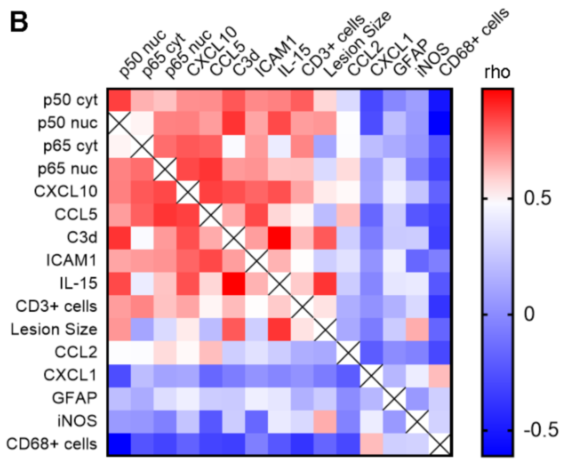

Supplementary Figure 8. Correlation of *in situ* astroglial expression of p50/65 NF-κB with NF-κB downstream target genes, infiltrating $CD3^+$ T cells, $CD68^+$ cells and lesion size. (**A**) Table of p-values (red indicates $p<0.05$) and Pearson rho (green indicates $r>0.7$) for computed Pearson correlation coefficients. All values passed the D'Agostino-Pearson omnibus normality test for Gaussian distribution. (**B**) Pearson rho values (red indicates $r>0.5$; blue indicates $r<0.5$) visualized by heatmap (red indicates $r>0.5$; blue indicates $r<0.5$).

Supplementary Figure 9. Demographics of MS patients assessed by T2 FLAIR MRI.

	age/SD (years)	gender f/m	disease duration/SD (years)
Yale (n=91)	48.25 ± 11.32	3.13	14.30 ± 11.98
Turku (n=43)	48.65 ± 10.04	2.58	14.18 ± 9.60
Risk genotype (n=78)	46.40 ± 11.42	2.39	13.69 ± 11.30
Prot. genotype (n=56)	51.21 ± 9.55	4.09	15.06 ± 11.19

Supplementary Figure 10.

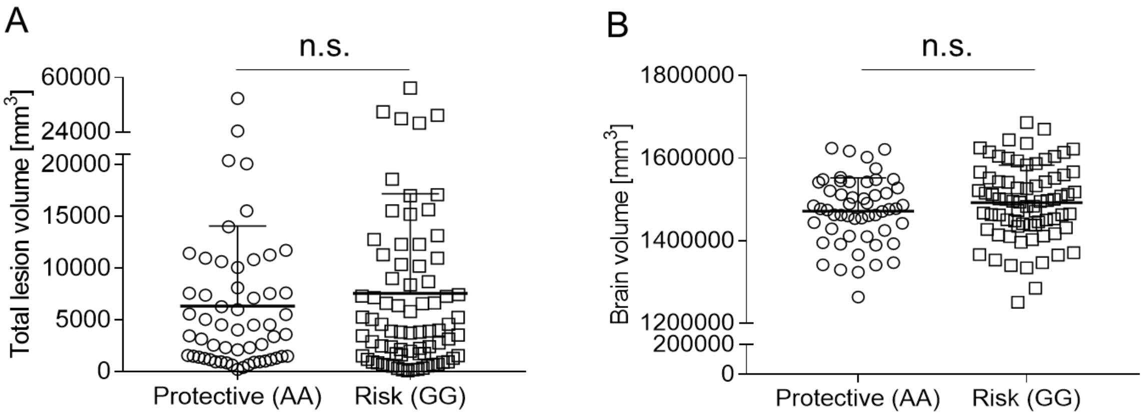

Supplementary Figure 10. Effect of the rs7665090 risk variant on total lesion load and brain volume in MS patients, measured by FLAIR and T1 MRI. (**A**) Total lesion volumes on FLAIR imaging of 78 (rs7665090^{GG}) and 56 (rs7665090^{AA}) MS patients. (**B**) Total brain volumes on T1 imaging of 74 (rs7665090^{GG}) and 53 (rs7665090^{AA}) MS patients. Data in (**A**) and (**B**) represent means + s.d..

Supplementary Figure 11. Antibodies used for immunohistochemistry, FACS and Western blot

Target	Antibody Type	Source	Catalog Number	Concentration
**Bright-field immunohistochemistry**				
MBP (myelin basic protein)	Rat (mAb)	Millipore Sigma	MAB386	1:500
CD68	Mouse (mAb)	Dako	M0876	1:500
CD3	Rabbit (polyAb)	Dako	A0452	1:150
**Fluorescent immunohistochemistry & Western Blot**				
GFAP	Chicken (polyAb)	Covance	PCK-591P	1:600
GFAP	Mouse (mAb)	Cell Signaling Technology	3670S	1:5000
CD68	Mouse (mAb)	Dako	M0876	1:100
NF-κB p50	Rabbit (mAb)	Abcam	ab32360	1:100
NF-κB p65	Rabbit (polyAb)	Novus Biologicals	NBP 2-24541	1:100
CCL5 (Chemokine ligand 5)	Rabbit (polyAb)	Novus Biologicals	NBP 1-19769	1:100
CCL2 (Chemokine ligand 2)	Rabbit (polyAb)	LS Bio	LS-B10540	1:100
CXCL10 (C-X-C motif chemokine10)	Rabbit (polyAb)	LS Bio	LS-C312561	1:50
CXCL1 (C-X-C motif chemokine1)	Rabbit (polyAb)	Novus Biologicals	NBP1-51188	1:100
IL-15	Mouse (mAb)	Abcam	ab55276	1:100
C3d Complement (N-Term)	Rabbit (mAb)	AntibodiesOnline	ABIN870581	1:100
ICAM1 (intercellular adhesion molecule 1)	Rabbit (polyAb)	Thermo-Fisher	PA5-27189	1:50
iNOS (inducible nitric oxide synthase)	Rabbit (polyAb)	Novus Biologicals	NB120-15203	1:100
CD31	Mouse (mAb)	Novus Biologicals	NBP2-818	1:50
Actin	Goat (polyAb)	Santa Cruz	sc-1616	1:10000
**Flow Cytometry**				
NF-κB p65 (pS529) Alexa Fluor 647	Mouse (mAb)	BD Biosciences	558422	1:5
STAT3 (pY705) Pacific Blue™	Mouse (mAb)	BD Biosciences	560312	1:5
IκBα (L35A5) Alexa Fluor 488	Mouse (mAb)	Cell Signaling Technology	5743	1:50
GLAST APC	Mouse (mAb)	Miltenyi	130-095-181	1:5
GFAP PE	Mouse (mAb)	BD Biosciences	561483	1:500

Supplementary Figure 12.

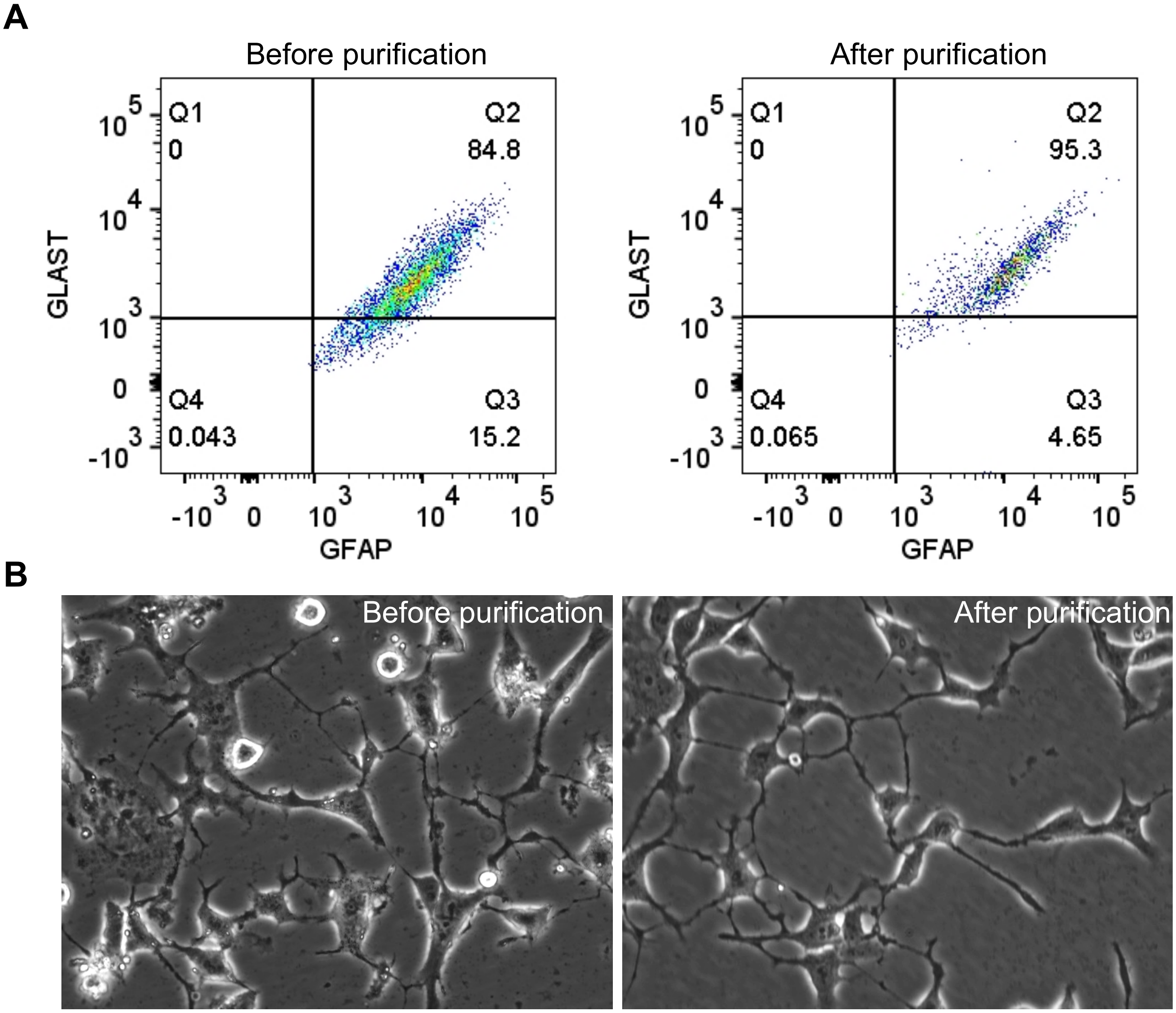

Supplementary Figure 12. Purification of iPSC-derived astrocytes with magnetic beads coupled to anti-GLAST antibodies. Quality control of the purification process by flow cytometry. (**A**) Before purification, 84.8% of astrocytes were GFAP/GLAST double-positive; after purification, 95.3% were double-positive. (**B**) Phase-contrast microphotographs of live cell cultures show a high degree of purity of cells with typical astroglial morphology before and after purification.
